# Dataset for compression ignition engine fuelled with corn oil methyl ester biodiesel

**DOI:** 10.1016/j.dib.2019.104683

**Published:** 2019-10-18

**Authors:** D. Balaji, T. Mathevan Pillai, K. Gnanasekaran, M. Balachandar, T.S. Ravikumar, S. Sathish, Ravishankar Sathyamurthy

**Affiliations:** a Department of Mechanical Engineering, Hindustan Institute of Technology and Science, Chennai, 603103, Tamil Nadu, India; b Department of Automobile Engineering, Hindustan Institute of Technology and Science, Chennai, 603103, Tamil Nadu, India; c Mechanical Power Engineering Department, Faculty of Engineering, Tanta University, Egypt

**Keywords:** Corn oil methyl ester, Engine, Performance, Biodiesel

## Abstract

The present data study deals with the experimental analysis of performance, emission and combustion characteristics of CI engine fuelled with corn oil methyl ester biodiesel blend as alternative fuel. A two-step trans esterification process is used to produce the biodiesel. Furthermore, a characteristics study was carried out on the extracted corn oil methyl ester biodiesel blends over conventional fuel. Three different fuel blends namely B10, B20 and B30 were chosen and the performance, emission and combustion characteristics of these are compared to that of conventional diesel fuel. Eddy current dynamometer is used load the engine from no load to maximum load condition. Using AVL DiGAS 444 N gas analyser and AVL 346 smoke meter the emissions and smoke opacity of the fuel blends and diesel were measured respectively. The experimental performance, emission and combustion data's were presented.

**Table:** Specifications Table

Subject	IC Engine
Specific subject area	Biodiesel, performance, emission, combustion
Type of data	Figures, Tables, Graphs
How data were acquired	Using computerized Kirloskar 4 stroke, DI-CI diesel engine is used to measure the engine performance, cylinder pressure and heat release. AVL DiGAS 444 N gas analyser and AVL 346 smoke meter, the emissions and smoke opacity is measured.
Data format	Raw and tabulated
Description of data collection	Using two stage trans esterification method, the pure corn oil is tp produce biodiesel. Based on ASTM standards, the physio-chemical properties were measured. Three different blends of biodiesel namely B10, B20 and B30 were used in DI-CI diesel engine.
Data source location	Sri Venkateswara College of Engineering, Sriperambadhur, Tamil Nadu, India
Data accessibility	With the article
Related research article	Liu, W., Lu, G., Yang, G., & Bi, Y. (2019). Improving oxidative stability of biodiesel by *cis*-trans isomerization of carbon-carbon double bonds in unsaturated fatty acid methyl esters. *Fuel*, *242*, 133–139. Saravankumar, P. T., Suresh, V., Vijayan, V., & Godwin Antony, A. (2019). Ecological effect of corn oil biofuel with SiO2 nano-additives. *Energy Sources, Part A: Recovery, Utilization, and Environmental Effects*, 1–8.

**Table:** 

**Value of the Data** • The data set provided shows the applicability of corn oil methyl ester at different blending ratio with diesel fuel in internal combustion engine. • The present data can be used to study the effect of blending biodiesel in different ratio and compare the fuel properties, combustion, emission and performance characteristics. • Researchers working on alternative fuels can use the present data for the comparative analysis

## 1 Data

**Fig. 1:**
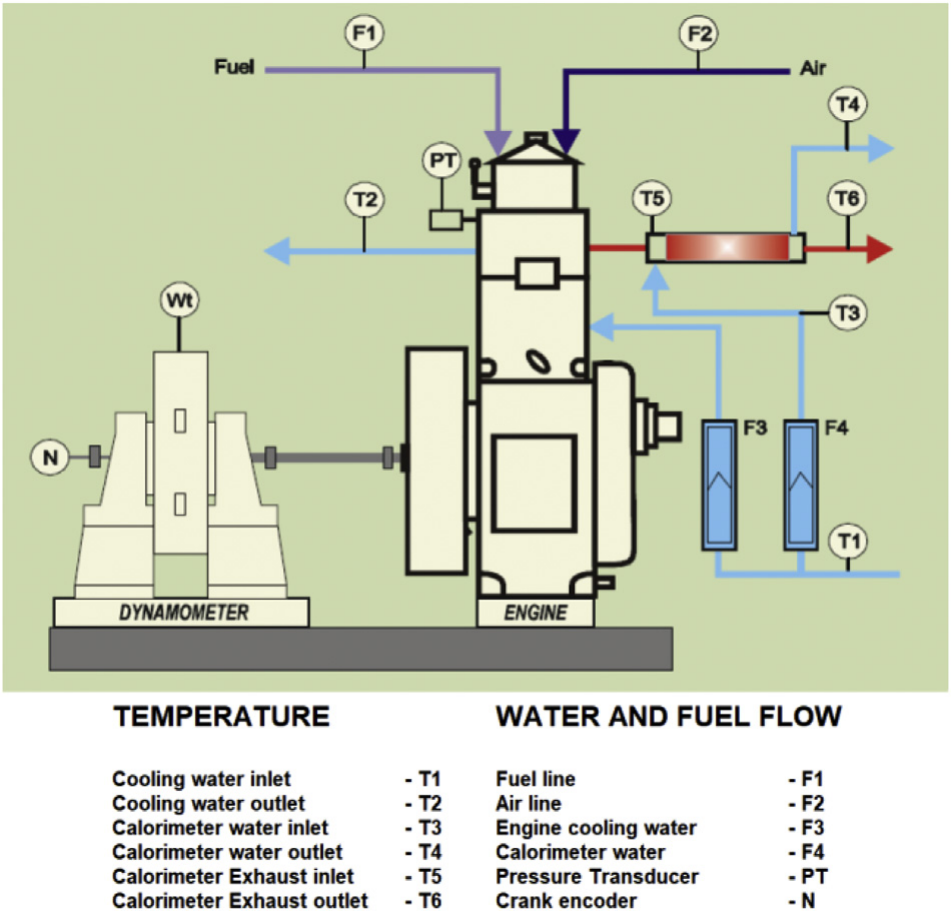
Schematic diagram of experimental setup.

**Fig. 2:**
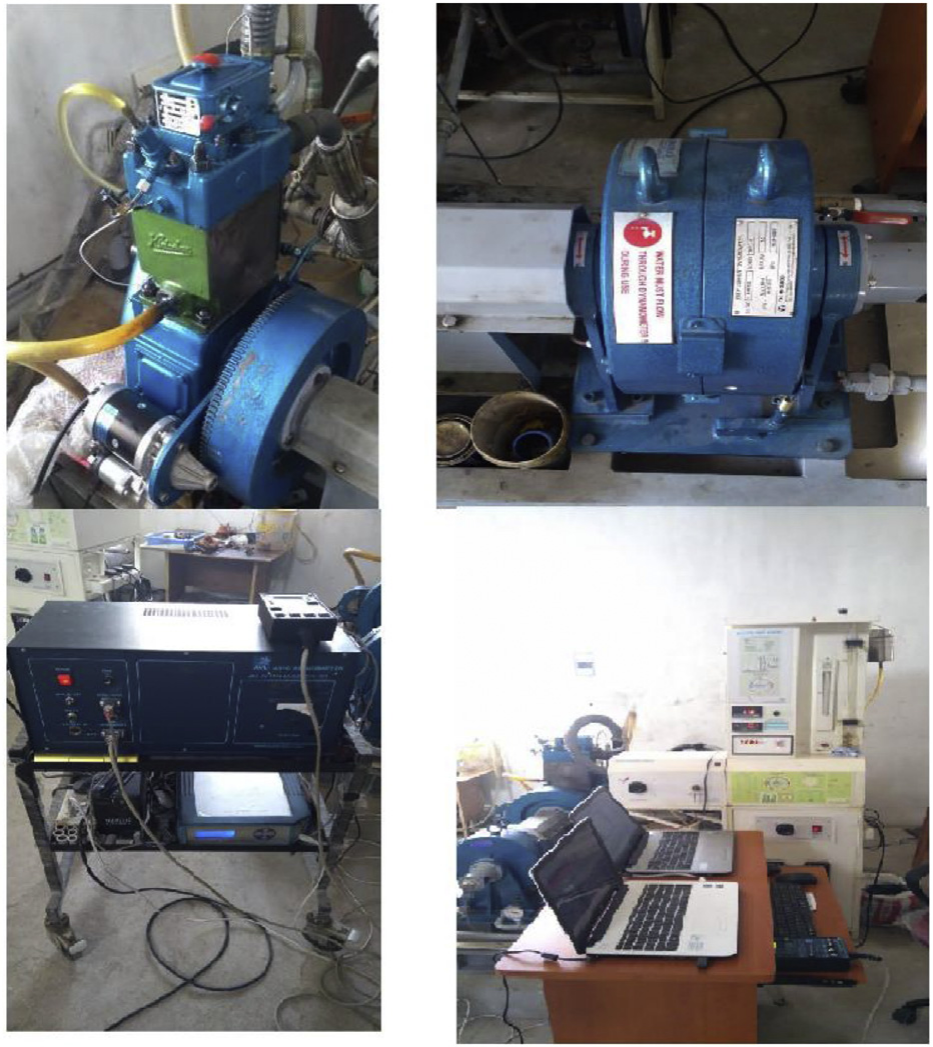
Photograph of experimental test rig, dynamometer, smoke meter with gas analyser and computerized test rig.

**Fig. 3:**
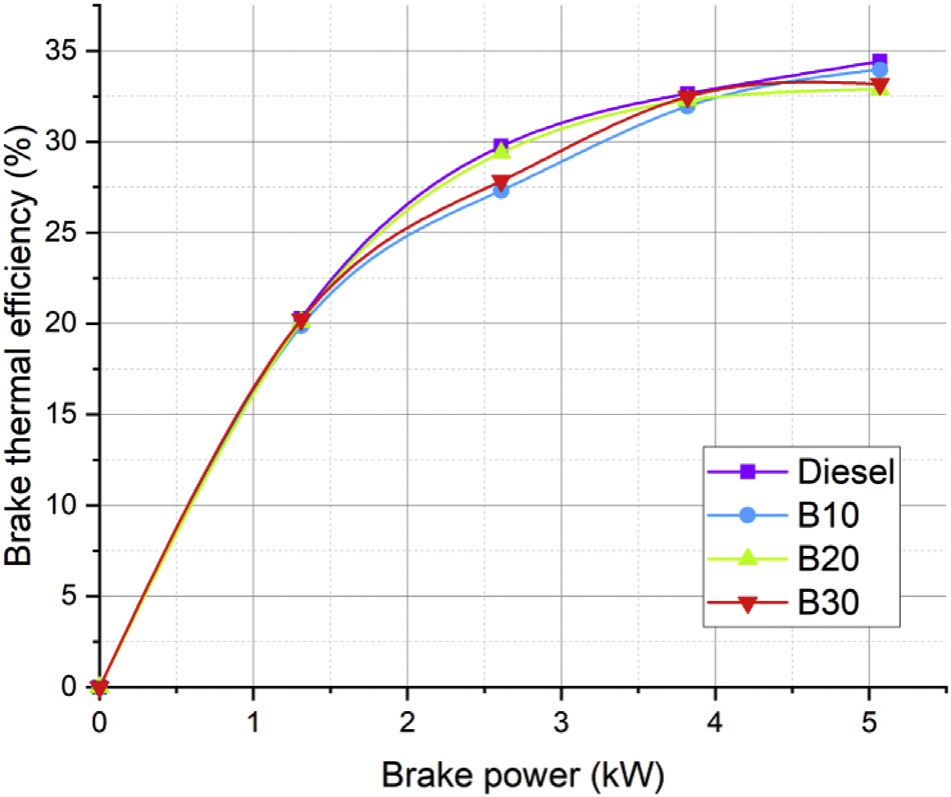
Variation in brake thermal efficiency of prepared fuel blends at different load condition.

**Fig. 4:**
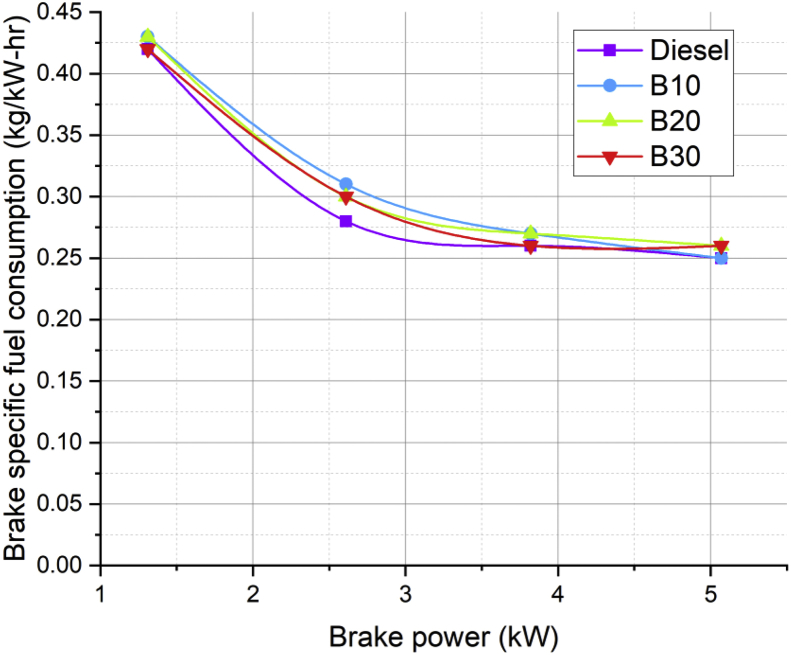
Variation in brake specific fuel consumption of prepared fuel blends at different load condition.

**Fig. 5:**
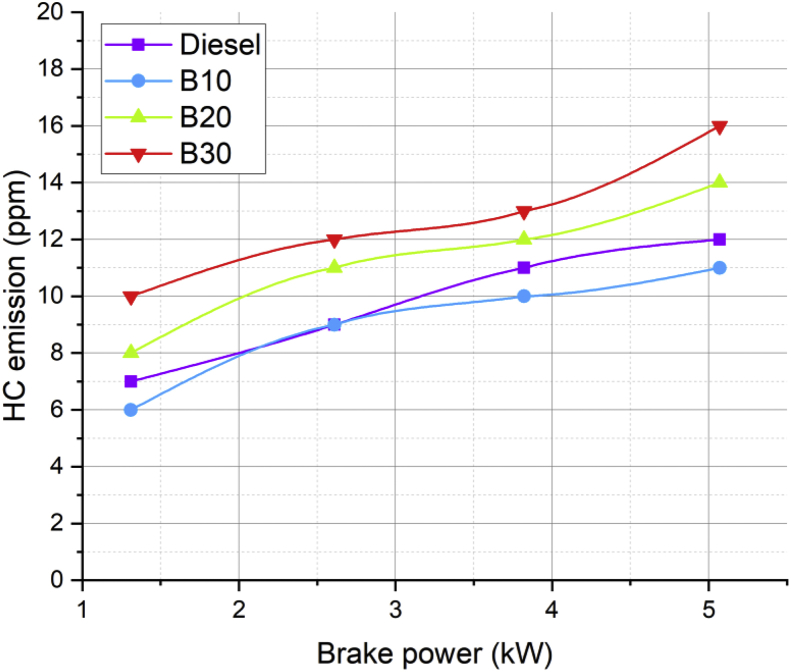
Variation in HC emissions of prepared fuel blends at different load condition.

**Fig. 6:**
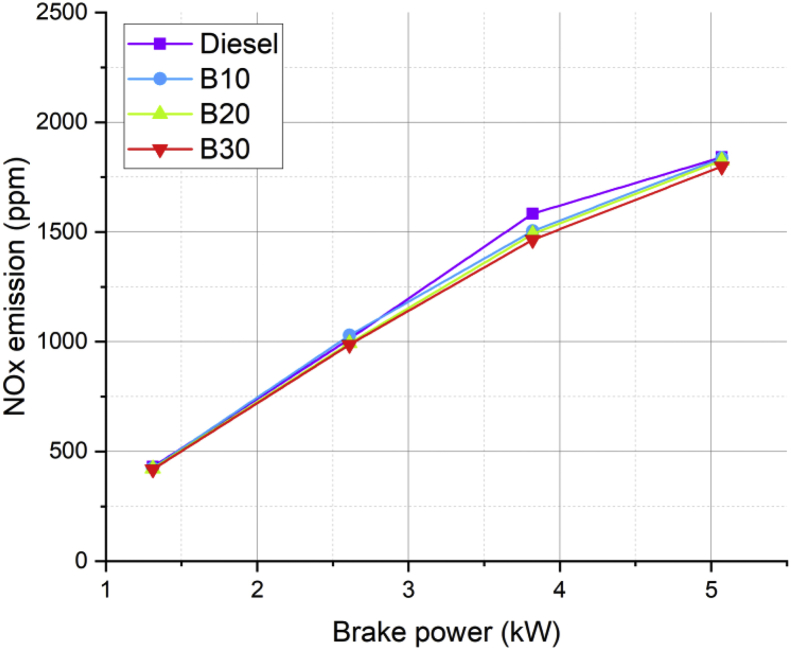
Variation in NOx of prepared fuel blends at different load condition.

**Fig. 7:**
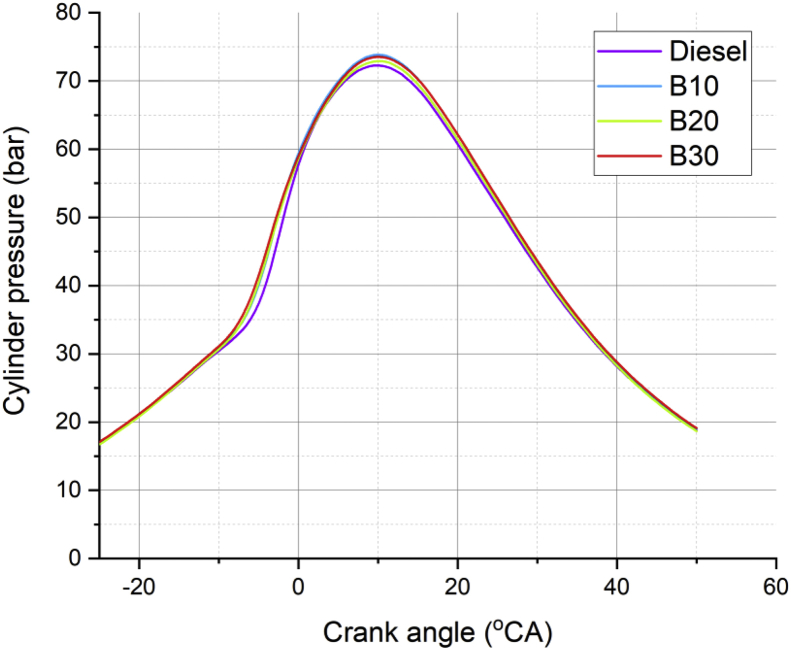
Variation in cylinder pressure developed inside the chamber for prepared fuel blends.

**Fig. 8:**
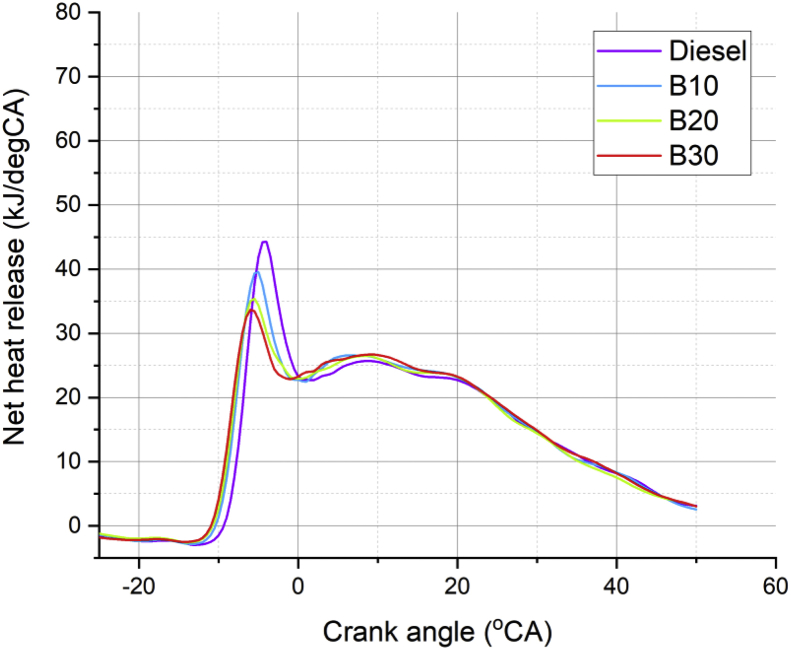
Variation in heat release rate of prepared fuel blends.

Lot of researchers have carried out different experiments on vegetable oil as an alternative fuel in compression ignition engine [1,2]. In the present data analysis various experiments were carried out on a single cylinder diesel engine fuelled with diesel, and blends of corn oil methyl ester biodiesel (B10, B20, and B30) to assess the engine performance and behavior over combustion and emission characteristics. The schematic diagram and experimental photograph of the test rig is shown in Fig. 1, Fig. 2 respectively. Based on the ASTM standards, the fuel properties such as kinematic viscosity, density, calorific value, flash point, fire point, and specific gravity of the fuel (B10, B20, B30, B100 and conventional diesel) and tabulated in Table 1. Table 2 shows the quantity of corn oil methyl ester and diesel for different blends of biodiesel. The detailed specification of engine used in the present study is tabulated in Table 3. The variations on the brake thermal efficiency of the engine fuelled with diesel and biodiesel blends is shown in Fig. 3. Similarly, the data set for brake thermal efficiency produced by the experimental test rig for diesel and different blends of biodiesel at various loads is tabulated in Table 4. Fig. 4 shows the variations in the fuel consumption of diesel, B10, B20 and B30 biodiesel blends while Table 4 also shows the data's of specific fuel consumption for diesel and different blends of biodiesel. Fig. 5 shows the variation of HC emission from prepared fuel blends and compared to that of conventional diesel. The data's associated with emissions from engine in the form of hydrocarbons and NOx is tabulated in Table 5. Fig. 6 shows the variation of NOx for all loading condition of prepared fuel blends. It is seen that the formation of NOx is lower in biodiesel blends compared to that of conventional diesel fuel whereas, the unburnt hydrocarbons from the engine is higher with blends of biodiesel. Also, it is seen that the optimum biodiesel concentration obtained is B10 as the performance characteristics of the blend (B10) is almost similar to conventional diesel fuel. Similarly, the variations in cylinder pressure and heat release for prepared fuel blends is shown in Fig. 7, Fig. 8 respectively. The data's associated with in cylinder pressure and net heat release rate are tabulated in Table 6, Table 7 respectively. It is observed from Fig. 7 that increasing the blend ratio of biodiesel decreased the cylinder pressure developed inside the cylinder. This is completely due to higher viscosity of fuel blend at higher blend ratio. Similarly, the rate of heat release is lower for biodiesel with higher blends as the calorific value of prepared biodiesel is lower as compared to that of diesel fuel (Fig. 8). The data set associated for different loads of engine on pressure developed and net heat release rate for diesel, B10, B20 and B30 are provided as a supplementary material.

**Table 1:** Properties of diesel and biodiesel blends.

Property	Diesel	B100	B10	B20	B30
Density (kg/m^3^)	823	902	855	860	865
Calorific value (kJ/kg)	42000	36640	41914	41328	40742
Kinematic viscosity (cSt)	2.8	6.44	3.34	3.65	3.92
Flash point	56	145	40	44	49
Fire point	64	156	51	54	60
Specific gravity	0.825	0.904	0.857	0.862	0.867

**Table 2:** Preparation of blends with diesel.

Blend	Fuel quantity (liters)	COME (ml)	Diesel (ml)
B10	1	100	900
B20	1	200	800
B30	1	300	700

**Table 3:** Engine specification.

Make	Kirloskar
Type	single cylinder 4 stroke water cooled diesel engine
Bore diameter	87.5 mm
Stroke length	110 mm
Compression Ratio	17.5:1
Combustion Chamber	Piston with hemispherical bowl
Maximum rated Speed	1500 rpm
Maximum operating Power	5.2 kW

**Table 4:** Data's for brake thermal efficiency for different biodiesel blends.

Load (%)	BP (kW)	Brake Thermal Efficiency (%)				Brake Specific fuel Consumption (kg/kW-hr)			
		Diesel	B10	B20	B30	Diesel	B10	B20	B30
25 (%)	1.31	20.27	19.88	20.1	20.23	0.42	0.43	0.43	0.42
50 (%)	2.61	29.75	27.31	29.39	27.84	0.28	0.31	0.3	0.3
75 (%)	3.82	32.65	31.95	32.28	32.48	0.26	0.27	0.27	0.26
100 (%)	5.07	34.42	33.98	32.9	33.17	0.25	0.25	0.26	0.26

**Table 5:** Emission data's (HC and NOx) for diesel and different biodiesel blends.

BP (kW)	HC emissions (ppm)				NOx emission (ppm)			
	Diesel	B10	B20	B30	Diesel	B10	B20	B30
1.31	7	6	8	10	430	425	422	419
2.61	9	9	11	12	1013	1029	993	987
3.82	11	10	12	13	1584	1505	1491	1465
5.07	12	11	14	16	1841	1837	1826	1799

**Table 6:** Data's of cylinder pressure developed using different blends of biodiesel.

Crank angle (θ)	Cylinder pressure developed using different blends of biodiesel (bar)			
	B10	B20	B30	Diesel
–25	17.005625	16.715	17.101875	16.821875
–24	17.775	17.485	17.864375	17.57625
–23	18.575	18.284375	18.654375	18.365
–22	19.405	19.11125	19.471875	19.184375
–21	20.26375	19.964375	20.3175	20.03125
–20	21.146875	20.844375	21.1925	20.903125
–19	22.05	21.75125	22.0975	21.7975
–18	22.975	22.684375	23.0325	22.713125
–17	23.9275	23.645	23.996875	23.650625
–16	24.908125	24.633125	24.9875	24.61
–15	25.910625	25.64125	25.998125	25.589375
–14	26.92375	26.655625	27.020625	26.583125
–13	27.93375	27.665	28.045625	27.578125
–12	28.930625	28.665625	29.06625	28.55875
–11	29.913125	29.661875	30.08625	29.516875
–10	30.8975	30.675625	31.133125	30.455625
–9	31.938125	31.77	32.286875	31.393125
–8	33.15625	33.08	33.71	32.379375
–7	34.758125	34.823125	35.6275	33.53625
–6	37.00375	37.23625	38.2275	35.096875
–5	40.084375	40.42125	41.525625	37.375625
–4	43.9475	44.209375	45.3	40.613125
–3	48.231875	48.209375	49.179375	44.755625
–2	52.4325	52.035	52.830625	49.37125
–1	56.184375	55.493125	56.0975	53.850625
0	59.390625	58.56125	58.994375	57.750625
1	62.12375	61.266875	61.59625	60.950625
2	64.485625	63.646875	63.956875	63.545
3	66.5475	65.745625	66.091875	65.683125
4	68.356875	67.588125	67.989375	67.474375
5	69.940625	69.170625	69.635625	68.9675
6	71.29375	70.48125	71.015625	70.178125
7	72.38875	71.51625	72.103125	71.11375
8	73.1925	72.273125	72.880625	71.77875
9	73.680625	72.743125	73.350625	72.16875
10	73.84625	72.91875	73.521875	72.276875
11	73.69625	72.798125	73.401875	72.103125
12	73.2425	72.381875	72.999375	71.653125
13	72.503125	71.678125	72.326875	70.938125
14	71.506875	70.708125	71.401875	69.97625
15	70.288125	69.50375	70.245	68.79125
16	68.883125	68.105625	68.8825	67.413125
17	67.330625	66.559375	67.35125	65.878125
18	65.6675	64.908125	65.693125	64.22625
19	63.92375	63.18625	63.94625	62.49625
20	62.12375	61.4175	62.141875	60.718125
21	60.28625	59.616875	60.3025	58.91125
22	58.423125	57.793125	58.44125	57.08875
23	56.543125	55.951875	56.565	55.259375
24	54.655625	54.098125	54.679375	53.426875
25	52.768125	52.23625	52.79125	51.59375
26	50.88375	50.37	50.90875	49.765625
27	49.006875	48.505	49.04125	47.949375
28	47.148125	46.65125	47.198125	46.15125
29	45.320625	44.82375	45.386875	44.37875
30	43.534375	43.039375	43.61375	42.640625
31	41.795625	41.31	41.885	40.945
32	40.10875	39.639375	40.205625	39.299375
33	38.475625	38.025625	38.578125	37.709375
34	36.895	36.465625	37.003125	36.176875
35	35.365625	34.95875	35.4825	34.7025
36	33.889375	33.505625	34.020625	33.286875
37	32.47125	32.10625	32.620625	31.92875
38	31.1175	30.7625	31.283125	30.625625
39	29.83125	29.4775	30.006875	29.375625
40	28.609375	28.251875	28.788125	28.17875
41	27.44625	27.083125	27.621875	27.035625
42	26.33875	25.9675	26.504375	25.945625
43	25.284375	24.901875	25.434375	24.9075
44	24.276875	23.88375	24.410625	23.918125
45	23.309375	22.910625	23.429375	22.971875
46	22.379375	21.98	22.486875	22.063125
47	21.48625	21.090625	21.583125	21.188125
48	20.62875	20.24375	20.720625	20.34625
49	19.805625	19.44	19.899375	19.53875
50	19.01625	18.67625	19.11625	18.766875

**Table 7:** Data's of Net heat release for different blends of biodiesel.

Crank angle (θ)	Net HR dQn/dq for different blends of bio diesel (kJ/deg CA)			
	B10	B20	B30	Diesel
–25	–1.38	–1.2	–1.79	–1.47
–24	–1.56	–1.42	–1.94	–1.57
–23	–1.76	–1.6	–2.06	–1.74
–22	–2.02	–1.8	–2.15	–1.98
–21	–2.26	–1.95	–2.19	–2.21
–20	–2.36	–1.97	–2.19	–2.36
–19	–2.32	–1.9	–2.13	–2.41
–18	–2.2	–1.84	–2.06	–2.35
–17	–2.15	–1.9	–2.1	–2.28
–16	–2.28	–2.12	–2.25	–2.3
–15	–2.55	–2.41	–2.42	–2.48
–14	–2.79	–2.57	–2.47	–2.79
–13	–2.83	–2.47	–2.35	–2.96
–12	–2.52	–2.01	–1.85	–2.86
–11	–1.43	–0.63	–0.12	–2.48
–10	1.43	2.78	4.25	–1.43
–9	7.49	9.62	12	1.33
–8	16.94	19.49	21.77	7.67
–7	28.61	29.62	30.06	18.33
–6	37.18	35.05	33.7	31.82
–5	39.56	34.37	32.14	41.89
–4	34.52	30.11	28.15	44.25
–3	28.9	26.84	24.46	38.41
–2	25.1	25.02	23.24	31.24
–1	22.97	23.27	22.85	26.2
0	22.77	22.94	23.3	23.24
1	22.46	23.12	23.97	22.84
2	23.65	23.82	24.12	22.74
3	24.58	24.3	25.16	23.35
4	25.76	24.73	25.67	23.63
5	26.33	25.43	25.88	24.35
6	26.55	25.97	26.07	25.01
7	26.56	26.33	26.43	25.41
8	26.5	26.46	26.61	25.64
9	26.44	26.46	26.73	25.68
10	26.07	26.11	26.64	25.58
11	25.64	25.61	26.38	25.24
12	25.21	25.01	26	24.88
13	24.86	24.46	25.46	24.39
14	24.55	24.06	24.88	23.92
15	24.31	23.89	24.4	23.48
16	24.18	23.88	24.11	23.27
17	24.05	23.86	23.94	23.2
18	23.89	23.8	23.81	23.12
19	23.59	23.59	23.62	22.99
20	23.11	23.19	23.26	22.7
21	22.54	22.62	22.68	22.23
22	21.91	21.85	21.91	21.63
23	21.11	20.9	21.05	20.83
24	20.07	19.79	20.18	19.9
25	18.96	18.59	19.26	18.95
26	17.92	17.43	18.33	17.95
27	17.05	16.47	17.43	16.98
28	16.24	15.74	16.56	15.99
29	15.46	15.1	15.71	15.19
30	14.66	14.42	14.86	14.44
31	13.78	13.68	13.96	13.72
32	12.8	12.83	13.06	13.07
33	11.81	11.89	12.25	12.41
34	10.94	10.97	11.57	11.77
35	10.32	10.17	11.04	11.07
36	9.95	9.57	10.58	10.33
37	9.63	9.05	10.1	9.68
38	9.28	8.56	9.5	9.08
39	8.84	8.07	8.85	8.58
40	8.32	7.52	8.18	8.19
41	7.86	6.88	7.55	7.83
42	7.21	6.24	6.84	7.35
43	6.42	5.63	6.11	6.68
44	5.67	5.11	5.5	5.89
45	5.03	4.67	4.92	5.11
46	4.4	4.32	4.47	4.41
47	3.76	4.04	4.09	3.88
48	3.24	3.74	3.75	3.5
49	2.84	3.42	3.44	3.28
50	2.58	3.06	3.04	3.13

## 2 Experimental design, materials, and methods

### 2.1 Materials and methods

#### 2.1.1 Preparation and production of biodiesel

Initially, the crude oil is trans esterified using NaOH to remove the soap content. A two stage acid and alkali based trans esterification method is employed to produce the biodiesel as the moisture content and free fatty acid content has to be removed. During the acid based 200ml of crude corn oil is taken in a beaker and heated for 10 minutes to a temperature of 60 °C and 60ml of methanol is added. In addition, 2ml of H_2_SO_4_ is added to the mixture and the mixture is stirred using magnetic stirrer for 1 hr at 50 °C. Then the mixture is allowed to settle for 2 hrs. The mixture with two separated phases namely methanol formed in the bottom and water floating on the top surface is formed. After the acid based trans esterification is done, the mixture is added with 50 ml methanol during the base catalyst trans esterification. Again the mixture is heated to a temperature of 60 °C and agitation is carried out at 1000 rpm. Solution of NaOH in methanol was added with the pre-treated oil at room temperature thus allowing the reaction for a period of 2 hrs. Using a separating funnel, the mixture was allowed to settle for almost 24 hrs and the lower glycerol layer is taken out.

#### 2.1.2 Experimental setup and procedure

The engine used in the present study is a Kirloskar type single cylinder, vertical, water cooled four stroke diesel engine. Various industrial and agricultural sector use these stationary engine so as in the present study this engine is chosen and this engine can be operated even at higher cylinder pressures. Eddy current dynamometer is used to load the engine. Using AVL DiGAS 444 type gas analyser the pollutants such as carbon dioxide, carbon monoxide, NOx, HC were measured and the smoke opacity is measured using AVL 346 smoke meter.
